# Antisense PMO cocktails effectively skip *dystrophin* exons 45-55 in myotubes transdifferentiated from DMD patient fibroblasts

**DOI:** 10.1371/journal.pone.0197084

**Published:** 2018-05-17

**Authors:** Joshua Lee, Yusuke Echigoya, William Duddy, Takashi Saito, Yoshitsugu Aoki, Shin’ichi Takeda, Toshifumi Yokota

**Affiliations:** 1 Department of Medical Genetics, Faculty of Medicine and Dentistry, University of Alberta, Edmonton, AB, Canada; 2 Department of Veterinary Medicine, Nihon University, Fujisawa, Kanagawa, Japan; 3 Northern Ireland Centre for Stratified Medicine, Altnagelvin Hospital Campus, Ulster University, Londonderry, United Kingdom; 4 Department of Molecular Therapy, National Institute of Neuroscience, National Center of Neurology and Psychiatry, Kodaira, Japan; 5 The Friends of Garrett Cumming Research & Muscular Dystrophy Canada HM Toupin Neurological Science Research Chair, Edmonton, AB, Canada; University of Minnesota Medical Center, UNITED STATES

## Abstract

Antisense-mediated exon skipping has made significant progress as a therapeutic platform in recent years, especially in the case of Duchenne muscular dystrophy (DMD). Despite FDA approval of eteplirsen–the first-ever antisense drug clinically marketed for DMD–exon skipping therapy still faces the significant hurdles of limited applicability and unknown truncated protein function. In-frame exon skipping of *dystrophin* exons 45–55 represents a significant approach to treating DMD, as a large proportion of patients harbor mutations within this “hotspot” region. Additionally, patients harboring *dystrophin* exons 45–55 deletion mutations are reported to have exceptionally mild to asymptomatic phenotypes. Here, we demonstrate that a cocktail of phosphorodiamidate morpholino oligomers can effectively skip *dystrophin* exons 45–55 *in vitro* in myotubes transdifferentiated from DMD patient fibroblast cells. This is the first report of substantive exons 45–55 skipping in DMD patient cells. These findings help validate the use of transdifferentiated patient fibroblast cells as a suitable cell model for *dystrophin* exon skipping assays and further emphasize the feasibility of *dystrophin* exons 45–55 skipping in patients.

## Introduction

Duchenne muscular dystrophy (DMD) is a lethal, progressive myopathy affecting approximately 1 in every 3600–5000 male births and is caused by deleterious mutations in the *dystrophin* (*DMD*) gene [1–4]. Mutations in *DMD* can also cause another milder form of muscular dystrophy known as Becker muscular dystrophy (BMD) [5]. Typically, DMD arises from out-of-frame mutations (~65% of patients) while BMD generally arises from in-frame mutations (~82% of patients) [6–8].

The observation that truncated dystrophin protein arising from in-frame mutant *DMD* transcripts could still maintain partial functionality–as in the case of BMD–helped provide the rationale for a therapeutic approach involving splice modulation via synthetic polymers. Antisense oligonucleotides (AOs) are chemically modified nucleic acids which can hybridize to pre-mRNA and can affect splicing and protein synthesis [9, 10]. By utilizing AOs, mutation-carrying exons and flanking exons can be selectively removed or “skipped” from the final messenger transcript, restoring the reading frame and producing a truncated protein which may retain some functionality–in essence, exon skipping could convert a DMD phenotype to a BMD phenotype [9, 11, 12]. Several *in vitro* and *in vivo* studies have now demonstrated the feasibility of antisense-mediated exon skipping in *DMD* [13–18] and the first-ever clinical AO drug for treating DMD, eteplirsen (Exondys 51), was approved by the FDA in 2016 [19].

Exon skipping as a therapy for treating DMD is not without its challenges. The first major drawback is the requirement for specifically-targeted AO sequences for any given exon. This means a great deal of time and money needs to be spent in evaluating individual AO sequences to address a wide range of patient mutation patterns. Another big challenge is determining the stability and functionality of the truncated proteins [20, 21]. A solution to the specific issue of validating oligo sequences for accommodating multiple mutation patterns is using analytical software algorithms to predict AO exon skipping efficiency, as has been described [22]. In conjunction with *in silico* predictive tools, another possible avenue to circumventing these issues is multi-exon skipping of *DMD* exons 45–55. First, in terms of applicability, a large proportion of all DMD patients (~47%) harbor mutations within this “hot-spot” region of exons 45–55, and up to ~63% of DMD patients with deletion mutations could benefit from skipping exons 45–55 [23, 24]. Multi-exon skipping of exons 45–55 would also address the issue of unknown truncated protein stability/function, as patients exhibiting this particular pattern of deletion mutation present with exceptionally mild symptoms or are asymptomatic [23–25].

As more AOs are required to bind to the same transcript to ensure maintenance of the open reading frame, dosage levels must be sufficiently high enough to facilitate skipping multiple exons while avoiding overt toxicity. While several AO chemistries have been designed and tested [9, 26], one of the most promising antisense chemistries developed is the phosphorodiamidate morpholino oligomer (morpholino or PMO). The stability, safety, and tolerability of PMOs have been well-documented [27–29], even at high doses [30, 31], and it is worth noting that the clinically-utilized eteplirsen is a PMO chemistry [32]. PMOs could therefore address issues regarding the potential toxicity of AO cocktails as they could be delivered at concentrations high enough to facilitate exons 45–55 skipping while avoiding toxic effects. Our group has demonstrated this previously in a dog model of DMD, using a cocktail of PMOs to effectively skip multiple exons *in vivo* without overt toxicity [14].

The first step toward clinical utility of any AO drug is the demonstration of its efficacy *in vitro*; thus, the establishment of a suitable *in vitro* exon skipping assay for determining *dystrophin* exon skipping is essential. To this end, muscle cell types (myoblasts and myotubes) expressing dystrophin are typically used. However, the collection of patient muscle tissue via biopsy is highly invasive, requires specialized equipment and preservation techniques [33], and yields only a small amount of what are often poorly proliferative cells [34]. Myotubes converted from fibroblasts via *MYOD1* transduction express dystrophin at levels sufficient to determine the effectiveness of AOs at facilitating *DMD* exon skipping and protein rescue [13] and represent an alternative approach to biopsied patient muscle tissue at evaluating exon skipping efficiency *in vitro*.

In this study, we tested the exons 45–55 skipping efficacy of PMO sequences designed using a predictive software tool by transfecting PMO cocktails into transdifferentiated DMD patient myotubes harboring deletion mutations amenable to reading frame correction via exons 45–55 skipping. We observed a dose-dependent production of exons 45–55 skipped transcripts as well as the rescue of dystrophin protein in DMD patient cells. This is the first-ever demonstration of robust dystrophin exons 45–55 skipping in transdifferentiated patient cells.

## Materials and methods

### Cell culture

Human DMD patient fibroblast cells harboring out-of-frame deletion mutations of *dystrophin* exons 45–50 (ID: GM05017) and exons 46–50 (ID: GM05162), as well as healthy human fibroblasts (ID: GM23815) were originally obtained in 2012 from the Coriell Institute for Medical Research (Camden, NJ, USA). DMD fibroblasts and human embryonic kidney cells (HEK293T; Cedarlane, ON, Canada) were cultured in DMEM/F-12 growth media (Invitrogen) containing 10% fetal bovine serum (FBS) and 0.5% penicillin/streptomycin and stored in a CO_2_ incubator at 37°C. For myotube differentiation, FACS-sorted fibroblasts were cultured in DMEM/F-12 (Invitrogen) containing 2% horse serum, 1X ITS Liquid Media Supplement (Sigma-Aldrich), and 0.5% penicillin/streptomycin.

### MYOD1 transduction and cell sorting

A pRetroX-IRES-ZsGreen1 expression vector (Clontech) containing the human *MYOD1* coding sequence, a pVSV-G envelope vector, and a gap-pol expression vector were prepared as described previously [13]. These vectors were co-transfected into HEK 293T cells via standard calcium phosphate transfection. Viral supernatant was collected after 48–72 h incubation and added to 70–80% confluent DMD fibroblast cells (40 mL viral supernatant in a T225 flask) along with 8 μg/ml polybrene (Sigma-Aldrich). DMD fibroblasts were incubated for 24 h at 37°C, then the viral supernatant was replaced with fresh growth media and cells were incubated for an additional 48 h. Cell sorting via FACS was performed on a FACS Area III flow cytometry system (BD Bioscience) by the Faculty of Medicine and Dentistry’s Flow Cytometry Core at the University of Alberta. Sorted ZsGreen-positive cells were seeded at 1x10^5^ cells in 500 μl of total volume into each well of a 12-well collagen-coated plate and incubated in growth media for 24 h. Culture media was then changed to differentiation media and cells were incubated until myotubes formed, with differentiation media being replaced every 2–3 d.

### Design and transfection of PMOs

The *in silico* design of 30-mer AOs targeting *DMD* exons 45–55 was performed using a predictive software algorithm developed by our group [22] and PMOs were synthesized by Gene Tools (Oregon, USA). PMOs at 1, 3 or 10 μM each were transfected as a cocktail into differentiated DMD myotubes using 6 μM Endo-Porter transfection reagent (Gene Tools). Cells were incubated with PMO for 48 h and then media was changed to fresh media and cells were incubated an additional 72 h before harvesting for analysis.

### RT-PCR analysis and sequencing

Total RNA was collected from cells using Trizol (Invitrogen) and 200 ng of total RNA was used for analyzing exons 45–55 skipping efficiency via SuperScript III One-Step RT-PCR System with Platinum Taq DNA Polymerase (Invitrogen). Primers for dystrophin: FWD GACAAGGGCGATTTGACAG; REV TCCGAAGTTCACTCCACTTG. Primers for GAPDH: FWD TCCCTGAGCTGAACGGGAAG; REV GGAGGAGTGGGTGTCGCTGT. PCR products were electrophoresed on a 1.5% agarose gel and bands were excised using a Wizard® SV Gel and PCR Clean-Up kit (Promega). Sanger sequencing of excised bands was performed by The Applied Genomics Core at the University of Alberta.

### Immunocytochemistry

Cells were fixed with 4% paraformaldehyde (PFA) for 5 min at room temperature, then permeabilized and blocked with 0.5% Triton X-100 and 10% goat serum for 20 minutes at room temperature. Cells were incubated in primary antibody for 1 h at room temperature at a 1:30 ratio for anti-desmin (ab8592, Abcam), anti-dystrophin (NCL-DYS1, Novocastra), and anti-myosin heavy chain (NCL-MHCf, Novocastra). Cells were incubated with 1:500 anti-rabbit or mouse IgG (H+L) highly cross-adsorbed secondary antibody, Alexa Fluor 594 (Invitrogen). SlowFade Gold Antifade Mountant with DAPI (Invitrogen) was added and cells were stored at 4°C.

## Results

### Patient mutation analysis and exon skipping approach

**Fig 1:**
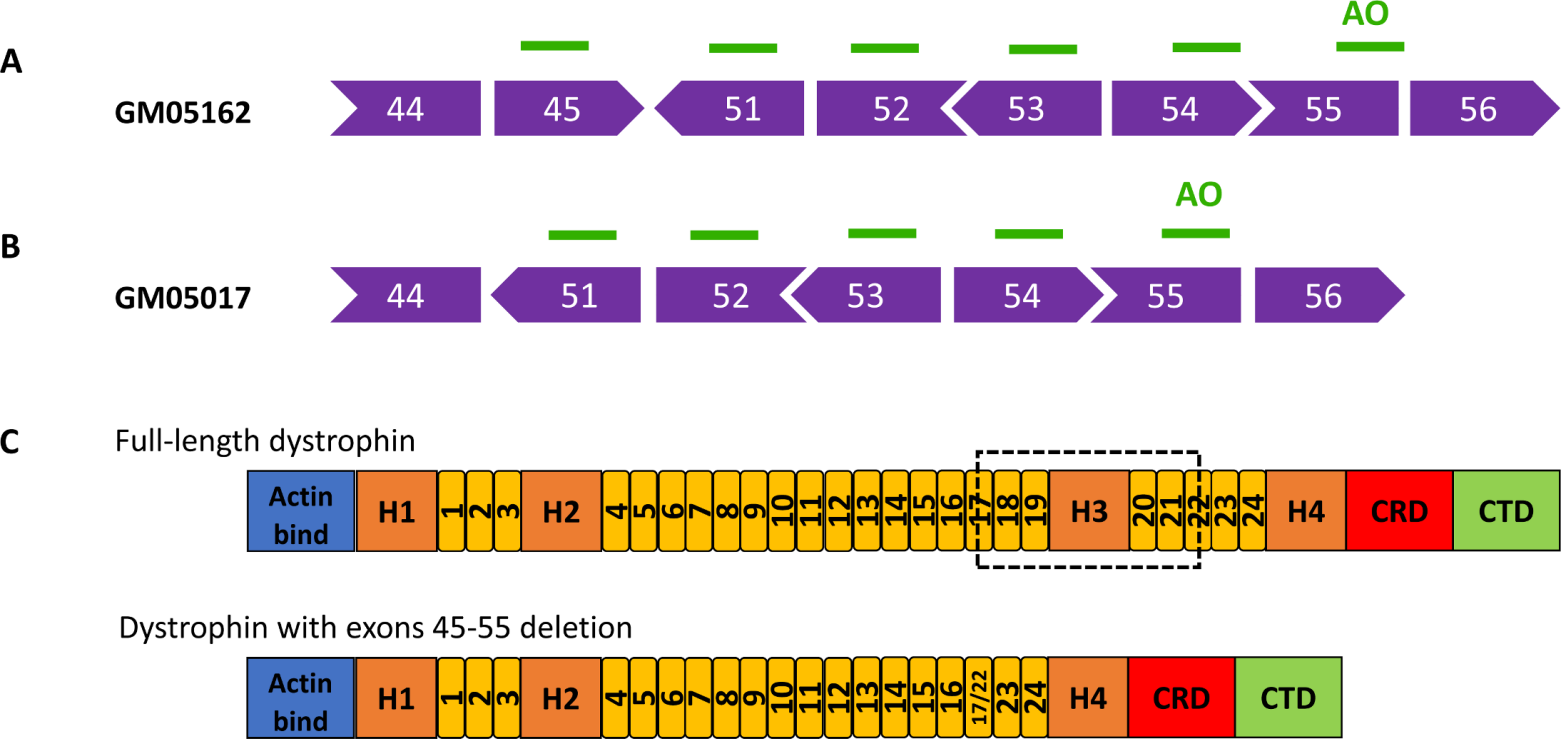
Mutation analysis and exon skipping approach. (A) Cell line GM05162 harbors an out-of-frame deletion mutation of *dystrophin* exons 46–50, which requires skipping 6 exons via PMOs to correct the reading frame. Sides of schematic boxes represent the codon phase. (B) Cell line GM05017 harbors an out-of-frame deletion mutation of *dystrophin* exons 45–50, which requires skipping 5 exons via PMOs to correct the reading frame. (C) Structure of full-length dystrophin and exons 45-55-deleted dystrophin. The truncated dystrophin generated by exons 45–55 skipping contains a hybrid rod repeat (yellow bars) of rods 17 and 22. Actin bind, actin-binding domain; H1-4, hinge domain 1–4; CRD, cysteine-rich domain; CTD, C-terminal domain.

DMD fibroblast cell lines GM05162 and GM05017 supplied by the Coriell Institute were originally sampled from two clinically affected males aged 13 and 12, respectively. Both individuals exhibited progressive muscle weakness and were wheelchair bound before or at age 10. GM05162 harbors a deletion of *dystrophin* exons 46–50, resulting in an out-of-frame product which requires a cocktail of 6 PMOs and skipping of exons 45, 51–55 to restore the reading frame (**Fig 1A**). GM05017 contains an out-of-frame deletion of exons 45–50, requiring a cocktail of 5 PMOs and skipping of exons 51–55 (**Fig 1B**). Based on these mutation patterns, we utilized our exon skipping predictive tool [22] to calculate the expected exon skipping efficiencies for PMO sequences of 30-mer length covering all possible target sites for the corresponding exons. PMO sequences used are described in **Table 1**. For our oligo design, we also considered the binding free energy values between AOs and selected oligos with >-9 ΔG **(Table 2)**. The in-frame skipping of dystrophin exons 45–55 produces a truncated product containing a hybrid rod repeat that is known to retain similar function to the full-length protein (**Fig 1C**).

**Table 1:** PMO sequences used for exons 45–55 skipping.

Exon Target	Distance from acceptor splice site	Sequence (5’ to 3’)	Predicted Skipping (%)
**Ex45**	**Ac9**	**GACAACAGTTTGCCGCTGCCCAATGCCATC**	**76.2**
**Ex51**	**Ac5**	**AGGTTGTGTCACCAGAGTAACAGTCTGAGT**	**73.0**
**Ex52**	**Ac24**	**GGTAATGAGTTCTTCCAACTGGGGACGCCT**	**90.1**
**Ex53**	**Ac9**	**GTTCTTGTACTTCATCCCACTGATTCTGAA**	**73.9**
**Ex54**	**Ac42**	**GAGAAGTTTCAGGGCCAAGTCATTTGCCAC**	**62.0**
**Ex55**	**Ac0**	**TCTTCCAAAGCAGCCTCTCGCTCACTCACC**	**120.4**

**Table 2:** Binding free energies between PMOs used for exons 45–55 skipping.

	Binding Free Energy (ΔG) between PMOs					
Exon Target	45	51	52	53	54	55
**45**	-4.5	-6.7	-6.7	-2.9	-3.3	-6.2
**51**		-8.7	-5.4	-6.5	-5.9	-7.9
**52**			-4.2	-3.2	-7.2	-5.3
**53**				-6.2	-7.4	-2.1
**54**					-8.4	-5.0
**55**						-2.7

### *MYOD1* transduction of DMD fibroblasts and conversion to myotubes

**Fig 2:**
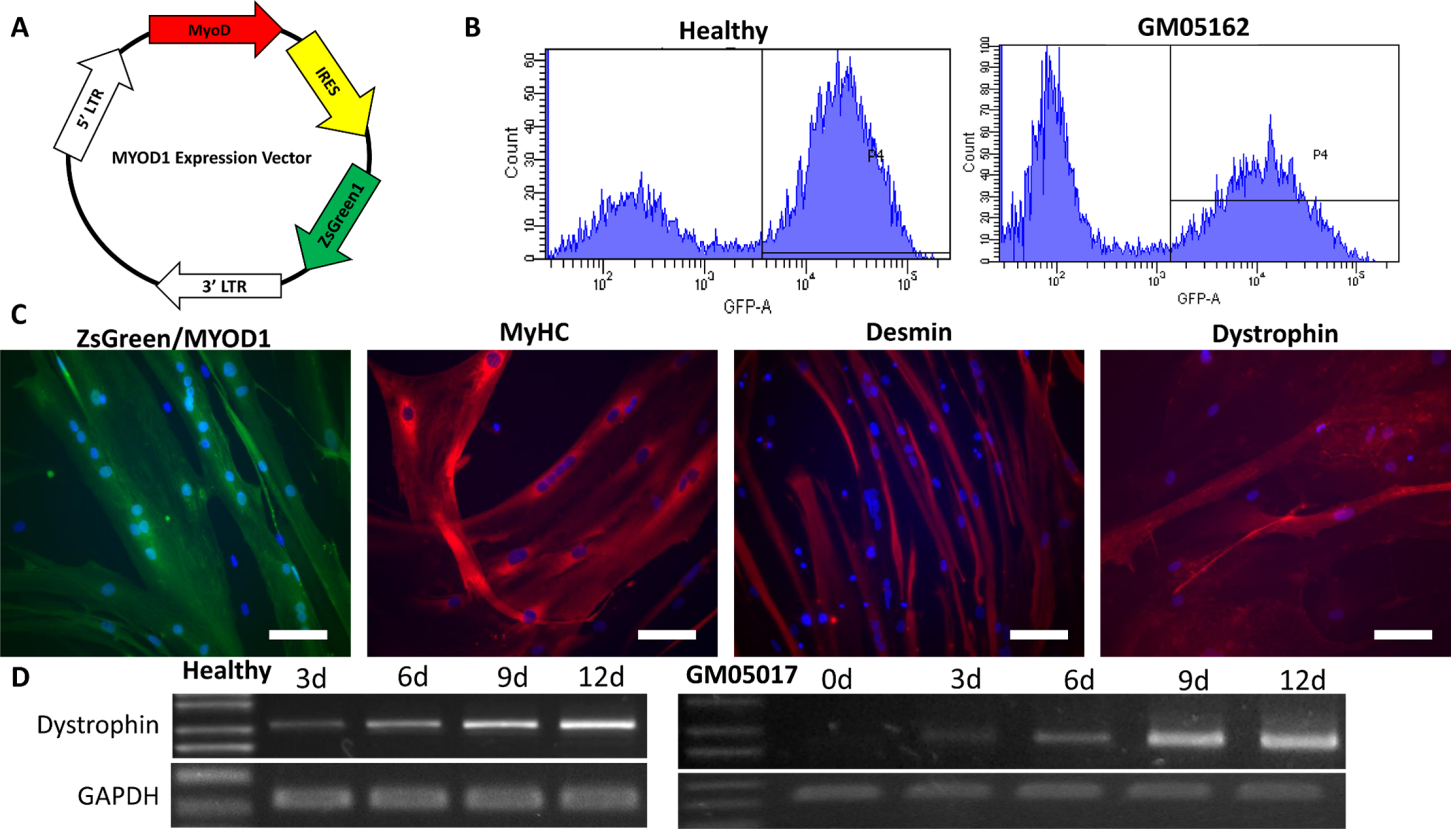
Transdifferentiation of DMD fibroblasts to myotubes. (A) Schematic diagram of *MYOD1* expression vector. (B) Histogram comparison of ZsGreen fluorescence signal vs cell number between healthy and DMD patient fibroblast cells transduced with *MYOD1* expression vector. Representative images shown, although results between either patient cell line were similar. (C) Immunocytochemistry of transduced fibroblasts following 18 d (MYOD1), 15 d (MyHC, myosin heavy chain), 18 d (desmin), and 24 d (dystrophin) differentiation, respectively. Pictured are results from a healthy cell line. Nuclei counterstained with DAPI. Scale bars: 100 μm. (D) RT-PCR time-course analysis of dystrophin expression in healthy and patient DMD transdifferentiated fibroblasts. Images are representative, with dystrophin expression observed in all transdifferentiated cell lines.

An expression vector containing ZsGreen and the human *MYOD1* coding sequence was delivered via retrovirus into human DMD patient fibroblast cells and healthy human fibroblasts (**Fig 2A**) [13, 35]. Following transduction, ZsGreen-positive cells were sorted via flow cytometry (**Fig 2B**) and seeded into collagen-coated 12-well plates. After adherence, cells were cultured in reduced-serum media to induce myogenic differentiation. Morphological examination showed that ZsGreen-positive *MYOD1*-transduced cells had become elongated and contained multiple nuclei (**Fig 2C**)–hallmarks of myotube morphology. To confirm successful transdifferentiation of fibroblasts to muscle cell type, we performed immunostaining for several markers of muscle identity. These cells expressed muscle-specific proteins myosin heavy chain and desmin, and healthy cells expressed dystrophin (**Fig 2D**). We then utilized a time-course expression assay to compare dystrophin mRNA expression between transdifferentiated healthy and patient cells. In both healthy and DMD patient cells, dystrophin mRNA expression was detectable by RT-PCR as early as 3 d after addition of differentiation media (**Fig 2E**).

### Antisense-mediated multi-exon skipping of *dystrophin* exons 45–55 in DMD patient cell lines using PMO cocktails

**Fig 3:**
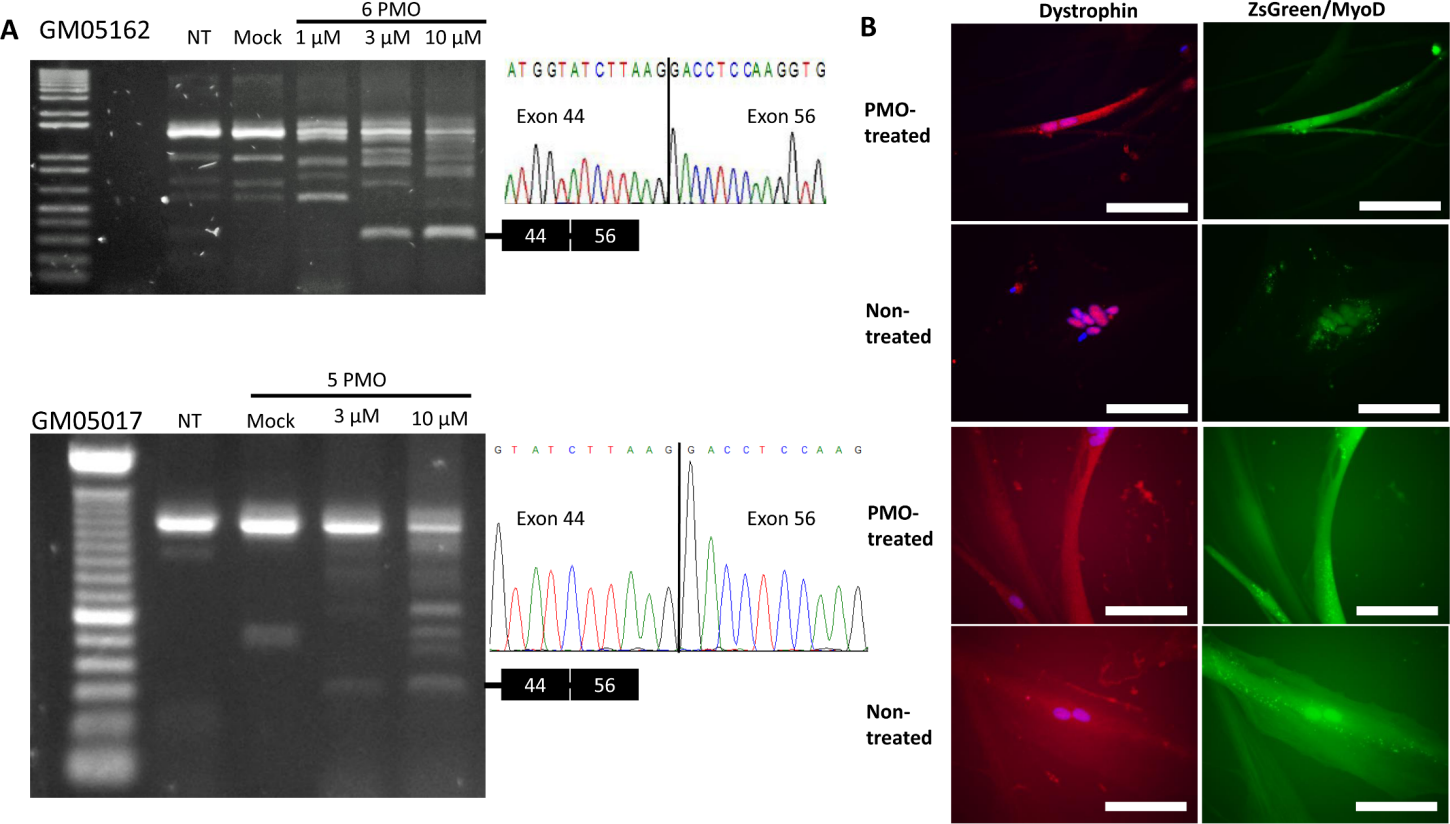
Multi-exon skipping of *dystrophin* exons 45–55 in transdifferentiated DMD patient cells. (A) RT-PCR for *dystrophin* following cocktail PMO transfection in transdifferentiated DMD patient cells. Cells were treated with 1, 3, or 10 μM each PMO. Expected molecular weight of *dystrophin* exons 45–55 skipped mRNA is 308 bp. (B) Representative immunocytochemistry of transduced DMD fibroblasts following PMO cocktail transfection. Nuclei counterstained with DAPI. Scale bars: 100 μm.

Based on respective mutation patterns, skipping of dystrophin exons 45–55 in cell line GM05162 requires a cocktail of 6 PMOs, while skipping of exons 45–55 in cell line GM05017 requires a cocktail of 5 PMOs (**Fig 1A and 1B**). Following transfection of PMO cocktails, RT-PCR analysis showed exon skipped products of the expected molecular weight in both cell lines in a dose-dependent manner (**Fig 3A**). Sanger sequencing of exon-skipped products revealed that the skipped products contained in-frame concatenations of *DMD* exons 44 and 56 (**Fig 3A**). Immunostaining showed some rescue of dystrophin protein in both PMO-treated cell lines (**Fig 3B**).

## Discussion

In this study, we demonstrated the feasibility of skipping *dystrophin* exons 45–55 *in vitro* using human DMD patients’ myotubes converted from fibroblasts. This is the first successful demonstration of robust, dose-dependent *dystrophin* exons 45–55 skipping in DMD patient cells. While earlier attempts at exons 45–55 skipping in patient cells were unsuccessful [36], our results emphasize the importance of AO sequence optimization and highlight the utility of *in silico* predictive screening for potential AO sequences.

This is also the first demonstration of exons 45–55 skipping in transdifferentiated cells, which underscores the suitability of using patient fibroblast cells as an alternative to cells obtained via muscle biopsy for evaluating exon skipping. This method of measuring exon skipping efficiency in transdifferentiated myotubes offers advantages to other assays, such as easy monitoring of *MYOD1* transduction efficiency via ZsGreen signal and effective induction of dystrophin expression, which can be difficult to induce [37].

Our group previously reported the efficacy of antisense-mediated *dystrophin* exons 45–55 skipping and rescue of dystrophin protein *in vivo* using a cocktail of vivo-morpholinos (vPMOs) in a mouse model of DMD [14, 15]. Before such new-generation antisense oligos can be effective and safe *in vivo* they require rigorous sequence optimization *in vitro*. Here, we emphasize an effective method for *in vitro* assessment of exon skipping efficacy in transdifferentiated human DMD patient cells which can pave the way for subsequent *in vivo* and clinical studies. By utilizing muscle cells obtained through fibroblast transdifferentiation, researchers can access an effective cell model for assessing the exon skipping ability of novel antisense chemistries and gene sequence targets while avoiding challenges associated with utilizing muscle harvested from patient biopsies, such as the limited availability and poor proliferative ability of patient muscle samples [34, 38].

Currently, the only clinically available exon skipping therapy is Sarepta’s exon 51 skipping drug, eteplirsen (Exondys 51). Notwithstanding FDA approval of the drug in 2016 [19], eteplirsen has remained surrounded by controversy, with concerns being raised as to its clinical efficacy [39]. Furthermore, exon 51 skipping is limited in its therapeutic applicability, with an estimated ~13% of all DMD patients being able to benefit from such an approach [40]. Several antisense-mediated exon skipping approaches are currently being evaluated across various clinical trials, targeting *DMD* exons 44 (NCT02958202), 45 (NCT02667483), 51 (NCT03375255), and 53 (NCT03167255); the respective therapeutic applicability of these targets is ~6%, ~8%, ~13%, and ~8% [40]. Notably, while the combined applicability of the aforementioned exon skipping approaches is ~35%, a single exon skipping approach targeting exons 45–55 would theoretically be amenable to ~47% of all DMD patients [24]. In addition to increased patient applicability, another advantage of skipping *DMD* exons 45–55 is that the resulting truncated protein is remarkably stable, as evidenced by patients harboring exons 45–55 deletion mutations who are either asymptomatic or display exceptionally mild symptoms [24].

In conclusion, our findings support the feasibility of future translation of the *dystrophin* exons 45–55 skipping approach into clinical practice for treating DMD. Potential hurdles such as possible side-effects of intermediate transcripts, toxicity assessment, and optimized PMO cocktail delivery will need to be addressed in future investigations. The development of a drug cocktail approach may also necessitate modification of existing regulatory body guidelines which currently consider individual AO sequences to be separate drugs. According to existing regulations, each individual component of a cocktail drug approach, including all possible combinations within that cocktail, would be required to undergo rigorous toxicological testing [41]. This creates significant barriers to the development of a drug cocktail approach which are both expensive and time-consuming to surmount.
